# Public health round-up

**DOI:** 10.2471/BLT.24.011024

**Published:** 2024-10-01

**Trauma in the Gaza Strip**

A man recovering from an injury in a refugee camp in Gaza's southern city of Rafah. According to a World Health Organization (WHO) analysis, released on 12 September, some 22 500 people have suffered life-changing injuries requiring long-term rehabilitation as a result of the ongoing conflict.

**Figure:**
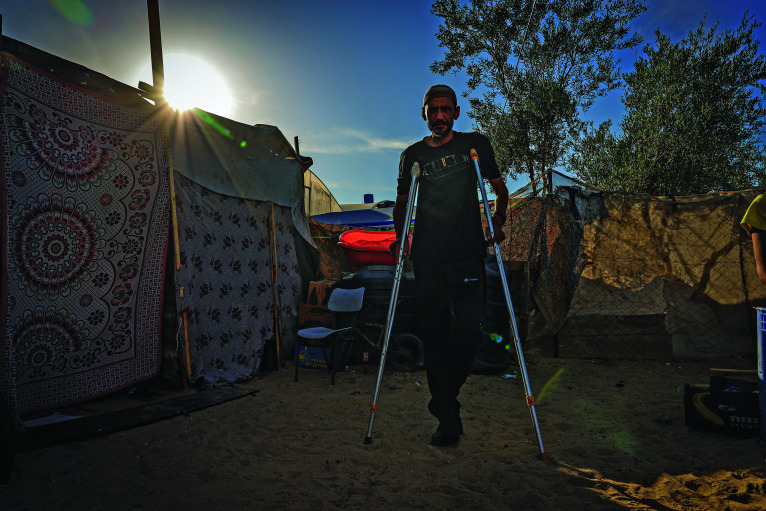
WHO

## Rehabilitation needs in the Gaza Strip

An estimated 22 500 people injured in the Gaza Strip due to the ongoing conflict have incurred life-changing injuries requiring long-term rehabilitation. This is according to a World Health Organization (WHO) analysis, released on 12 September.

*Estimating trauma rehabilitation needs in Gaza using injury data from emergency medical teams*, indicates that severe limb injuries (estimated to be between 13 455 to 17 550) are the main driver of rehabilitation needs. There have also been between 3105 and 4050 limb amputations. Spinal cord injuries, traumatic brain injuries and major burns have further contributed to the growing number of life-altering injuries.

“The huge surge in rehabilitation needs occurs in parallel with the ongoing decimation of the health system,” said Dr Richard Peeperkorn, WHO Representative in the occupied Palestinian territory. “Patients can’t get the care they need. Acute rehabilitation services are severely disrupted, and specialized care for complex injuries is unavailable, putting patients’ lives at risk.” 

The estimates in the analysis will be used by WHO and partners to plan for a surge in rehabilitation-related services and contribute to long-term health planning and policy-making.

https://bit.ly/4gnONEJ

## Polio vaccination in the Gaza Strip

The first round of a polio vaccination campaign in the Gaza Strip using novel oral polio vaccine type 2 (nOPV2) was brought to a successful close in September. The campaign was conducted by the Palestinian Ministry of Health in collaboration with WHO, the United Nations Children’s Fund (UNICEF), the United Nations Relief and Works Agency for Palestine Refugees (UNRWA) and partners, and was made possible by the implementation of humanitarian pauses in the fighting. It was also facilitated by the overwhelming response from parents who were willing to get their children immunized.

According to a 13 September WHO media briefing, as of that date, 552 451 children under 10 years of age had been vaccinated, 105 909 in the north, 195 722 in the middle and 250 820 in the south. A second round is due to be implemented in the following 4–6 weeks.

The campaign was launched in response to the detection of circulating variant poliovirus type 2 (cVDPV2) in environmental samples collected from central Gaza in June 2024. Four cases of children with acute flaccid paralysis (AFP), including one case of confirmed polio in a child who tested positive for cVDPV2, were also reported. Two of the reported cases tested negative for poliovirus. As of 12 September, laboratory results were pending regarding samples from the fourth AFP case.

https://bit.ly/3zoyK9d

## Addressing the Sudan crisis

WHO called on the international community to urgently act to end the extreme health and humanitarian crisis in Sudan. Some 25 million people require urgent humanitarian assistance there, having been forced to flee their homes due to the ongoing conflict and the destruction of essential infrastructure and services.

The appeal was made during a two-day visit to Port Sudan by WHO Director-General Tedros Adhanom Ghebreyesus and Regional Director Hanan Balkhy, which took place on 7 and 8 September. The WHO officials met with Sudanese leaders, and discussions centred on the devastating impact of the ongoing conflict and the critical need for unhindered humanitarian access to ensure that life-saving aid reaches all those in need.

“The international community has seemingly forgotten about Sudan and is paying little heed to the conflict tearing it apart, with serious repercussions for the region,” Director-General Tedros said.

https://bit.ly/4ejGWqa

https://bit.ly/3z5jXAh

## mpox vaccines and diagnostics

WHO prequalified the MVA-BN mpox vaccine, the first vaccine against mpox to be added to its prequalification list. Announced on 13 September, the prequalification is based on information submitted by the manufacturer Bavarian Nordic A/S and is expected to facilitate access to this vital product, reducing transmission and helping contain the outbreak.

WHO also requested that manufacturers of mpox in vitro diagnostics submit an expression of interest for emergency use listing.

WHO and partners established an access and allocation mechanism for mpox medical countermeasures, including vaccines, treatments and diagnostic tests. Developed in coordination with Member States, the mechanism is intended to increase access to these tools for people at highest risk and ensure that the limited supplies are used effectively and equitably.

In related news, UNICEF issued an emergency tender for the procurement of mpox vaccines on 31 August. The tender was issued in collaboration with the Africa Centres for Disease Control and Prevention, Gavi, the Vaccine Alliance, WHO, the Pan American Health Organization and other partners to help secure mpox vaccines for the countries hardest hit by the ongoing outbreak.

As of 8 September, some 25 237 suspected and confirmed cases and 723 deaths had been reported from outbreaks in 14 countries of the African Region.

https://bit.ly/3XHwAuE

## Cholera rising

Cholera is increasing worldwide, with WHO reporting a 13% rise in cases (535 321 in 2023, up from 472 697 in 2022) and a 71% increase in deaths (4007 in 2023, up from 2349 in 2022). Cases were reported in 45 countries – up from 44 the previous year.

The disease, spread through contaminated food and water, disproportionately affects communities with poor sanitation. However, factors such as conflict, climate change, inadequate water access, poverty and population displacement have fuelled the rise in outbreaks.

Many countries reported deaths outside of health facilities, signalling critical gaps in access to treatment. Large outbreaks occurred in Afghanistan, the Democratic Republic of the Congo, Malawi and Somalia, with Ethiopia, Haiti, Mozambique and Zimbabwe also heavily impacted.

The trend appears to be continuing in 2024, with 342 800 cases and 2400 deaths reported across 22 countries by August. WHO continues to view the global cholera risk as very high. Despite this, the Organization’s 50 million United States dollars (US$) appeal for cholera response in 2024 remains largely unfunded.

https://bit.ly/3XEEMvy

## Tackling antibiotic pollution

WHO published its first-ever guidance on wastewater and solid waste management for antibiotic manufacturing. The guidance sheds light on the important but neglected challenge of antibiotic pollution ahead of the United Nations General Assembly (UNGA) High-Level Meeting on antimicrobial resistance (AMR) scheduled to take place on 26 September 2024.

The emergence and spread of AMR caused by antibiotic pollution is contributing to the decline in effectiveness of antibiotics globally, including the medicines produced at manufacturing sites that are partly responsible for the pollution.

Antibiotic pollution levels are largely unregulated, and quality assurance criteria for manufacturing typically do not address environmental emissions. In addition, there is a lack of information provided to consumers on how to dispose of antibiotics when they expire or are unused.

https://bit.ly/3TsNXwF

**Cover photo**

A health worker conducts a community awareness session on mosquito net use in Sohbatpur, Balochistan province, Pakistan.

**Figure:**
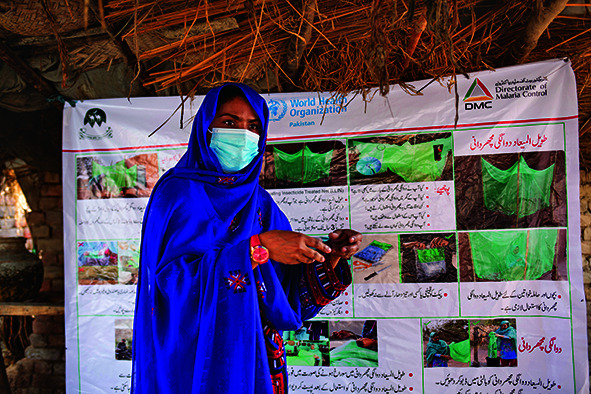
WHO/Panos Pictures/Saiyna Bashir

## Investigating new and re-emerging pathogens

WHO published a global framework to help countries investigate the origins of new and re-emerging pathogens. Published on 4 September with support from the Scientific Advisory Group for the Origins of Novel Pathogens, the framework aligns with the International Health Regulations (2005) and the One Health approach, and aims to prevent future outbreaks by halting transmission and reducing animal-to-human spillover.

The framework outlines six key areas for scientific investigation, ranging from early case investigations to identify exposure sources and pathogen characteristics, to genomic studies to trace pathogen evolution and distribution.

https://bit.ly/4eh1dMX

## African countries and partners invest in WHO

Some 14 African countries and health partners pledged over US$ 45 million to the WHO Investment Round, an initiative aimed at generating sustainable financing for the Organization.

The commitments were made on 27 August during the 74th session of the WHO Regional Committee for Africa which took place in the Republic of the Congo, with heads of state and government from across the continent underscoring the importance of investing in global health.

https://bit.ly/3Xmd8Cm

**Looking ahead**

13–15 October. World Health Summit. Berlin, Germany. https://bit.ly/3B1Iqqx

14–16 October. World Summit on Public Health and Health Sciences. Bern, Switzerland. https://bit.ly/3XGku4N

29–31 October. The Africa Health Tech Summit. Kigali, Rwanda. https://bit.ly/3B3Gr56

